# Active site architecture of coproporphyrin ferrochelatase with its physiological substrate coproporphyrin III: Propionate interactions and porphyrin core deformation

**DOI:** 10.1002/pro.4534

**Published:** 2023-01-01

**Authors:** Andrea Dali, Thomas Gabler, Federico Sebastiani, Alina Destinger, Paul Georg Furtmüller, Vera Pfanzagl, Maurizio Becucci, Giulietta Smulevich, Stefan Hofbauer

**Affiliations:** ^1^ Dipartimento di Chimica “Ugo Schiff” – DICUS Università di Firenze Sesto Fiorentino (FI) Italy; ^2^ Department of Chemistry Institute of Biochemistry, University of Natural Resources and Life Sciences Vienna Austria; ^3^ INSTM Research Unit of Firenze Sesto Fiorentino (Fi) Italy

**Keywords:** ferrochelatase, heme biosynthesis, resonance Raman, site‐directed mutagenesis, X‐ray crystallography

## Abstract

Coproporphyrin ferrochelatases (CpfCs) are enzymes catalyzing the penultimate step in the coproporphyrin‐dependent (CPD) heme biosynthesis pathway, which is mainly utilized by monoderm bacteria. Ferrochelatases insert ferrous iron into a porphyrin macrocycle and have been studied for many decades, nevertheless many mechanistic questions remain unanswered to date. Especially CpfCs, which are found in the CPD pathway, are currently in the spotlight of research. This pathway was identified in 2015 and revealed that the correct substrate for these ferrochelatases is coproporphyrin III (cpIII) instead of protoporphyrin IX, as believed prior the discovery of the CPD pathway. The chemistry of cpIII, which has four propionates, differs significantly from protoporphyrin IX, which features two propionate and two vinyl groups. These findings let us to thoroughly describe the physiological cpIII‐ferrochelatase complex in solution and in the crystal phase. Here, we present the first crystallographic structure of the CpfC from the representative monoderm pathogen *Listeria monocytogenes* bound to its physiological substrate, cpIII, together with the in‐solution data obtained by resonance Raman and UV–vis spectroscopy, for wild‐type ferrochelatase and variants, analyzing propionate interactions. The results allow us to evaluate the porphyrin distortion and provide an in‐depth characterization of the catalytically‐relevant binding mode of cpIII prior to iron insertion. Our findings are discussed in the light of the observed structural restraints and necessities for this porphyrin‐enzyme complex to catalyze the iron insertion process. Knowledge about this initial situation is essential for understanding the preconditions for iron insertion in CpfCs and builds the basis for future studies.

## Abstract 2

PDB Code(s): 8AT8 and 8AW7;

## 1 INTRODUCTION

Ferrochelatases are essential for heme *b* biosynthesis^1^ and their decreased activity has been reported to be associated with several diseases.^2^, ^3^ Differently from the “protoporphyrin‐dependent” (PPD) heme biosynthesis pathway, where the protoporphyrin ferrochelatase (PpfC) is active at the last stage of the biosynthetic process, coproporphyrin ferrochelatase (CpfC) catalyzes the penultimate reaction in the “coproporphyrin‐dependent” (CPD) heme biosynthesis pathway. Here Fe^2+^ ion is incorporated into coproporphyrin III (cpIII) to give Fe^3+^‐coproheme, which is afterwards decarboxylated by the coproheme decarboxylase enzyme, finally yielding protoheme.^1^, ^4^, ^5^ Diderm bacteria and eukaryotes predominantly use the PPD pathway, whereas monoderm bacteria utilize the CPD pathway. The oxidation of two pyrrole nitrogens and the insertion of ferrous iron into the porphyrin ring are catalyzed by homologous enzymes in both pathways (CPD and PPD). However, the substrates of CpfC and PpfC are cpIII and protoporphyrin IX (PPIX), respectively (Figure S1), which differ for the presence of propionates in positions 2 and 4 in the cpIII, which are vinyls in PPIX. Before the discovery of the CPD pathway in 2015,^4^ all the studies investigating ferrochelatases (fCs) involved in the CPD pathway were performed in the belief that these enzymes were part of the PPD pathway, and, therefore, protoporphyrin IX or other two‐propionate porphyrins (e.g., mesoporphyrin) were used as substrates instead of four‐propionate cpIII. Only more recently a biochemical investigation of the cpIII‐CpfC from the actinobacteria *Staphylococcus aureus* (*Sa*CpfC) in solution has been performed.^6^, ^7^ However, to the best of our knowledge, no structure of bacterial ferrochelatases in complex with the physiological cpIII substrate is available. Several X‐ray crystallographic structures have been determined for wild‐type and variant forms of *Bacillus subtilis* (*Bs*CpfC),^8^, ^9^, ^10^, ^11^, ^12^, ^13^ using either PPIX or the proposed transition state analog of the ferrochelatase‐catalyzed reaction *N*‐methylmesoporphyrin (N‐MeMP), and 2,4‐disulfonic acid deuteroporphyrin IX (dSDP) (similar to cpIII but with sulfonate groups replacing the propionate groups in position 2 and 4) as substrate. In all these structures the porphyrin macrocycle shows specific spatial arrangement and orientation within the active‐site pocket, different also from the substrate‐bound human ferrochelatase,^14^ the most extensively studied ferrochelatase among mammals. The human ferrochelatase is an inner mitochondrial membrane‐associated enzyme that possesses an essential [2Fe‐2S] cluster of yet unknown function within the active site and displays a methionine residue coordinating the metal center after iron insertion, in contrast to a tyrosine or phenylalanine in monomeric CpfCs.^4^

While previous studies provided valuable information concerning the overall structure together with the identification of iron binding and porphyrin deprotonation sites, essential details on the bacterial enzyme substrate binding, conversion, release, and regulation are still missing. The use of non‐physiological substrates to study the Gram‐positive ferrochelatases lead to wrong conclusions.^15^ In fact, due to the substantial structural differences between eukaryotic and monoderm ferrochelatases, conclusions concerning the catalytically‐relevant binding orientations of cpIII cannot be drawn, since the physiological substrate has two more propionates than protoporphyrin IX and, therefore, can interact differently with the protein moiety. Accordingly, the first crystallographic structure of CpfC with the physiological product (coproheme), recently obtained for *Listeria monocytogenes* (*Lm*CpfC), together with a detailed analysis of its stability and activity in solution, shows that specific H‐bond interactions are established between the polar amino acids in the active site and the four propionates of coproheme. These interactions govern the complex stability and binding specificity of the system.^16^, ^17^

We now present the first crystallographic structure of the native substrate of CpfCs, cpIII, bound to *Lm*CpfC. In addition, we report a comprehensive biochemical and spectroscopic analysis in solution for these systems. Thanks to this work, we determine unequivocally the catalytically‐relevant binding orientation of cpIII within the CpfC active‐site pocket, and uncover the role of specific residue side chains H‐bonding interactions with the porphyrin propionate for its stabilization.

## 2 RESULTS

### 2.1 Crystal structure

**FIGURE 1:**
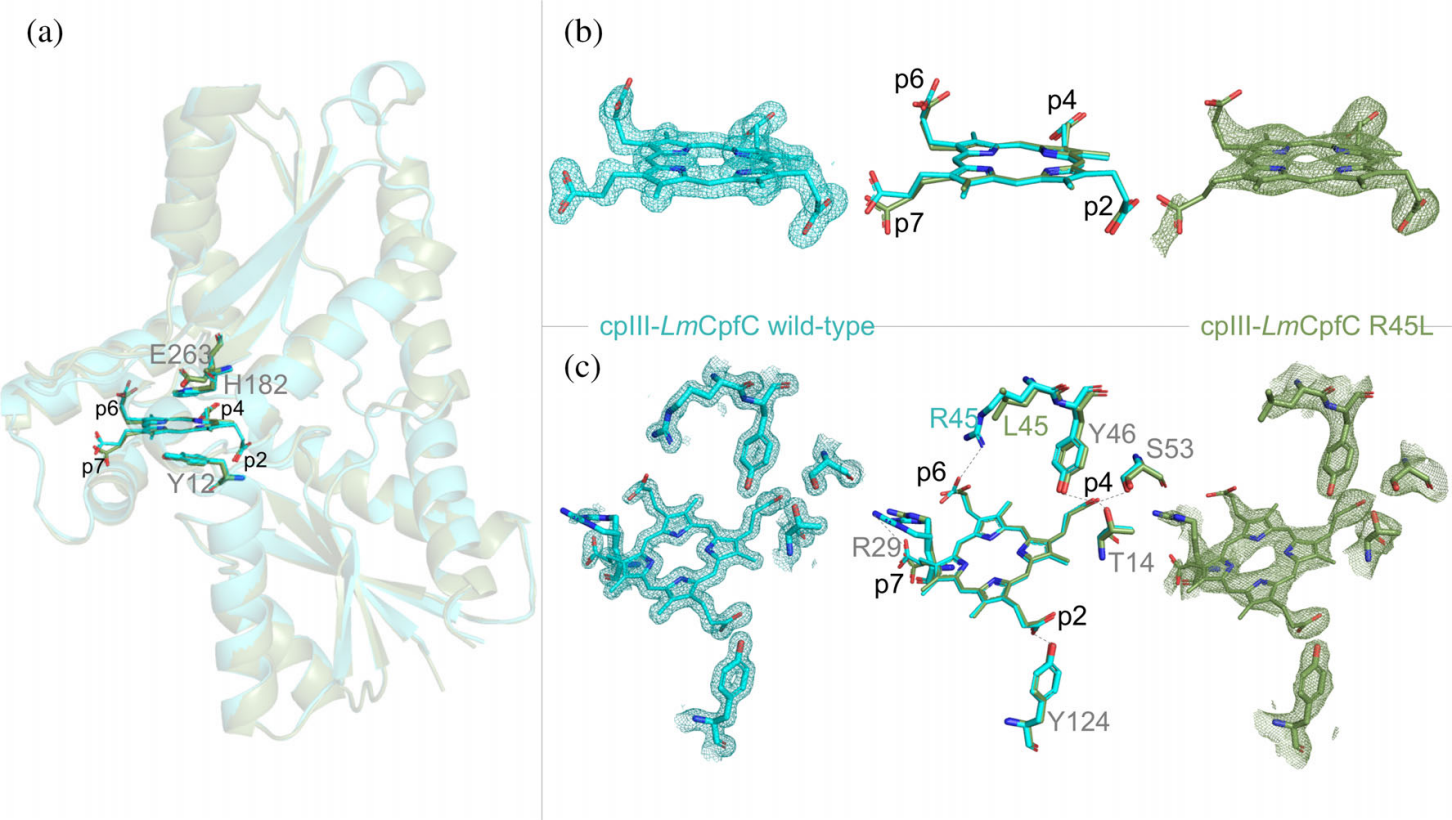
Structures of cpIII‐*Lm*CpfC wild‐type (cyan, PDB ID: 8AT8) and cpIII‐*Lm*CpfC R45L (olive, PDB ID: 8AW7). (a) Overlay of monomeric coproporphyrin ferrochelatases with bound substrates, and secondary structural elements are depicted as semi‐transparent cartoons and coproporphyrin III as well as distal H182, E263, and proximal Y12 are shown as sticks. (b) Stick representation of cpIII in *Lm*CpfC wild‐type and R45L including electron densities (2fofc maps, *σ* = 1.0), represented as meshes. (c) Representation of propionate interactions with amino acid residues in H‐bonding distance, sticks, and meshes as in (b)

For the first time, structural information of the physiological catalytic situation is available. The structures of *Lm*CpfC wild‐type (WT) and of the R45L variant were solved in complex with the natural substrate cpIII (Figure 1). The R45L variant was investigated since, lacking the H‐bonding with the propionate at position 6 (p6), highlights the importance of this propionate interaction in *Lm*CpfC. It helped to explain distinct spectral features observed by resonance Raman (RR) spectroscopy in solution, and allowed us the assignment of the propionate bending modes (see below).

Diffraction data for *Lm*CpfC wild‐type and for the R45L variant were collected to 1.51 Å and 2.64 Å resolution, respectively (Table 1). Structures were solved by molecular replacement using the apo‐*Lm*CpfC structure (PDB ID: 6RWV)^16^ and refined to *R*_free_ values of 0.1816 (cpIII‐*Lm*CpfC wild‐type) and 0.2112 (cpIII‐*Lm*CpfC R45L); for complete data collection and refinement statistics see Table 1. As for the product‐ (coproheme‐) bound *Lm*CpfC structure (PDB ID: 6SV3), the monomeric enzyme exhibits two ferredoxin‐like domains, each with a four‐stranded parallel β‐sheet flanked by α‐helices. In spite of less than 10% amino acid sequence identity between *Lm*CpfC or *Bacillus subtilis* CpfC and *Homo sapiens* ferrochelatase, the core regions of their crystallographic structures share a root mean square deviation of 2.4 Å.^18^ The X‐ray crystallographic structures for the dimeric human and *Saccharomyces cerevisiae* PpfCs and those for the monomeric *B. subtilis* and *B. anthracis* CpfCs revealed that PpfC and CpfC adopt a similar protein fold, which is also observed in all the structures solved for *Lm*CpfC (apo‐, coproheme‐, cpIII‐bound).^8^, ^19^, ^20^

**TABLE 1:** Data collection and refinement statistics

	*Lm*CpfC wild‐type (PDB ID: 8AT8)	*Lm*CpfC R45L (PDB ID: 8AW7)
Wavelength (Å)	0.97	0.97
Resolution range (Å)	36.78–1.51 (1.56–1.51)	32.7–2.64 (2.734–2.64)
Space group	P 1 21 1	P 1
Unit cell (Å, °)	37.63, 68.07, 63.04 90, 102.22, 90	37.70, 48.93, 52.47 92.40, 103.52, 109.05
Total reflections	96,285 (9671)	32,873 (3080)
Unique reflections	48,370 (4852)	9928 (985)
Multiplicity	2.0 (2.0)	3.3 (3.1)
Completeness (%)	99.11 (99.79)	98.68 (97.51)
Mean I/sigma(I)	13.42 (2.36)	17.93 (3.34)
Wilson B‐factor	17.58	50.88
R‐merge	0.02909 (0.2962)	0.06186 (0.309)
R‐meas	0.04114 (0.4189)	0.07466 (0.3872)
R‐pim	0.02909 (0.2962)	0.04102 (0.2283)
CC1/2	0.999 (0.854)	0.978 (0.314)
CC*	1 (0.96)	0.995 (0.691)
Reflections used in refinement	48,350 (4851)	9915 (981)
Reflections used for R‐free	2345 (232)	524 (52)
R‐work	0.1461 (0.2368)	0.1703 (0.2129)
R‐free	0.1816 (0.2570)	0.2112 (0.3311)
CC(work)	0.972 (0.917)	0.963 (0.892)
CC(free)	0.944 (0.852)	0.955 (0.815)
Number of non‐hydrogen atoms	2912	2565
Macromolecules	2542	2497
Ligands	127	54
Solvent	299	14
Protein residues	308	309
RMS (bonds)	0.011	0.005
RMS (angles)	1.06	0.69
Ramachandran favored (%)	97.06	96.09
Ramachandran allowed (%)	2.94	3.91
Ramachandran outliers (%)	0.00	0.00
Rotamer outliers (%)	0.00	0.38
Clashscore	2.55	6.63
Average B‐factor	24.92	59.22
Macromolecules	23.61	59.20
Ligands	26.38	61.16
Solvent	35.73	56.64
Number of TLS groups	6	3

*Note*: Statistics for the highest‐resolution shell are shown in parentheses.

A cleft, build by structural elements of both ferredoxin‐like domains, contains several conserved amino acid residues (distal H182 and E263; proximal Y12) and is the porphyrin binding site, where catalysis happens (Figure 1a), as already shown for CpfC from *Bacillus subtilis*.^11^ When bound to the enzyme, cpIII is distorted leading to a distinctly non‐planar geometry in both the wild‐type protein and the R45L variant (Figure 1b). Some uncertainty remains on the detailed orientation of the carboxyl groups of the more flexible propionate p6 and p7 in the structure of cpIII‐*Lm*CpfC R45L, due to weak electron densities. Nevertheless, the spatial orientation of the propionates towards the respective H‐bonding partners can be identified also in the R45L variant. In the wild‐type structure on the other hand, cpIII is very well resolved with clear electron densities for all groups.

In cpIII‐*Lm*CpfC, as well as in the coproheme‐bound structure, the porphyrin is oriented in the active site with the propionates 2 and 4 (p2 and p4) pointing to the inner core of the protein, p6 and p7 face towards the protein surface and are much more solvent exposed (Figure 1c).

We recently showed that in both cpIII and coproheme complexes with *Lm*CpfC the H‐bond interactions between the four propionates and six amino acids, Thr14, Arg29, Arg45, Tyr46, Ser53, and Tyr124, are fundamental to the porphyrin stabilization.^16^, ^17^ While the overall binding interactions between three propionate groups (p2, p4, p6) and the respective residues are identical for the product‐ (coproheme‐) bound *Lm*CpfC^16^ and the substrate‐ (cpIII‐) bound *Lm*CpfC (this work), the porphyrin distortion of the iron‐free cpIII and the iron‐loaded coproheme are significantly different. In the cpIII‐*Lm*CpfC structure, R29 is present in a split conformation (52% in H‐bonding distance to p7; 48% in the “out” conformation), which is not the case in the coproheme‐bound *Lm*CpfC structure nor in the coproporphyrin‐bound R45L structure where R29 is pointing towards p7. The “out” conformation of R29 was previously observed also in the structure of apo‐*Lm*CpfC (PDB ID: 6RWV).^16^ It has to be noted that disruption of this R29‐p7 interaction, by introducing the R29L variant, did not have a significant effect to its biochemical and biophysical properties in solution.^17^

**FIGURE 2:**
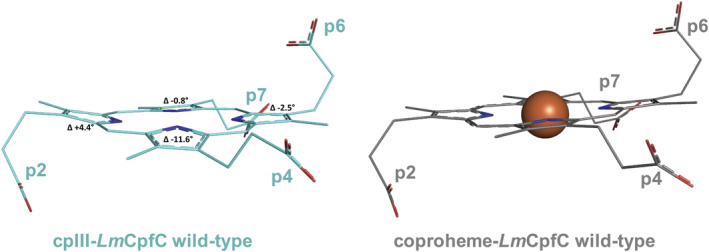
Representation of porphyrins from cpIII‐*Lm*CpfC (left, cyan lines, pdb‐code: 8AT8) and coproheme‐*Lm*CpfC wild‐type (right, gray lines, pdb‐code: 6SV3). Distortion angles of the pyrrole rings of coproporphyrin III in the PDB ID: 8AT8 are indicated compared to the coordinates of coproheme in PDB ID: 6SV3. The central iron atom in coproheme (iron coproporphyrin III) is shown as an orange sphere

In Figure 2 we compare the active site structures of the cpIII‐*Lm*CpfC and coproheme‐*Lm*CpfC WT complexes. These porphyrin conformations differ significantly. It can be clearly seen that in the cpIII‐*Lm*CpfC complex, pyrrole ring A is tilted up by approximately 4° with respect to pyrrole rings of the coproheme‐*Lm*CpfC complex (PDB ID: 6SV3), whereas pyrrole ring B (~12°) and pyrrole ring D (~1°) are tilted down. Pyrrole ring C is in plane. This porphyrin architecture is best described as a saddled‐like distorted porphyrin.^21^, ^22^, ^23^ On the other hand, the coproheme in the wild‐type structure (PDB ID: 6SV3) exhibits an almost completely planar porphyrin core, stabilized by the inserted central iron.

### 2.2 Coproporphyrin III bound to *Lm*CpfC: Effect of porphyrin binding into the pocket in solution

RR is widely applied to the study of heme‐containing proteins, having contributed significantly to the understanding of their structure–function relationship.^24^ In fact, the pioneer work by Spiro and coworkers in the 1970s showed that in the 1300–1700 cm^−1^ RR spectral region, the wavenumbers of the so‐called “core size marker bands” are inversely correlated with the size of the porphyrin core, reflecting the coordination, oxidation and spin states of the heme iron. These results opened the door to the assignment of heme protein coordination and spin states to either a 5‐coordinated (5c) high spin (HS), 6‐coordinated (6c) HS, or low spin (LS) state.^25^, ^26^, ^27^, ^28^, ^29^ Moreover, in 1988 the combination of site‐directed mutagenesis and RR study of recombinant cytochrome *c* peroxidase (CCP) cloned in *Escherichia coli* made it possible to highlight the heme pocket interactions and the role played by the conserved key residues important for enzymatic activity.^30^

**FIGURE 3:**
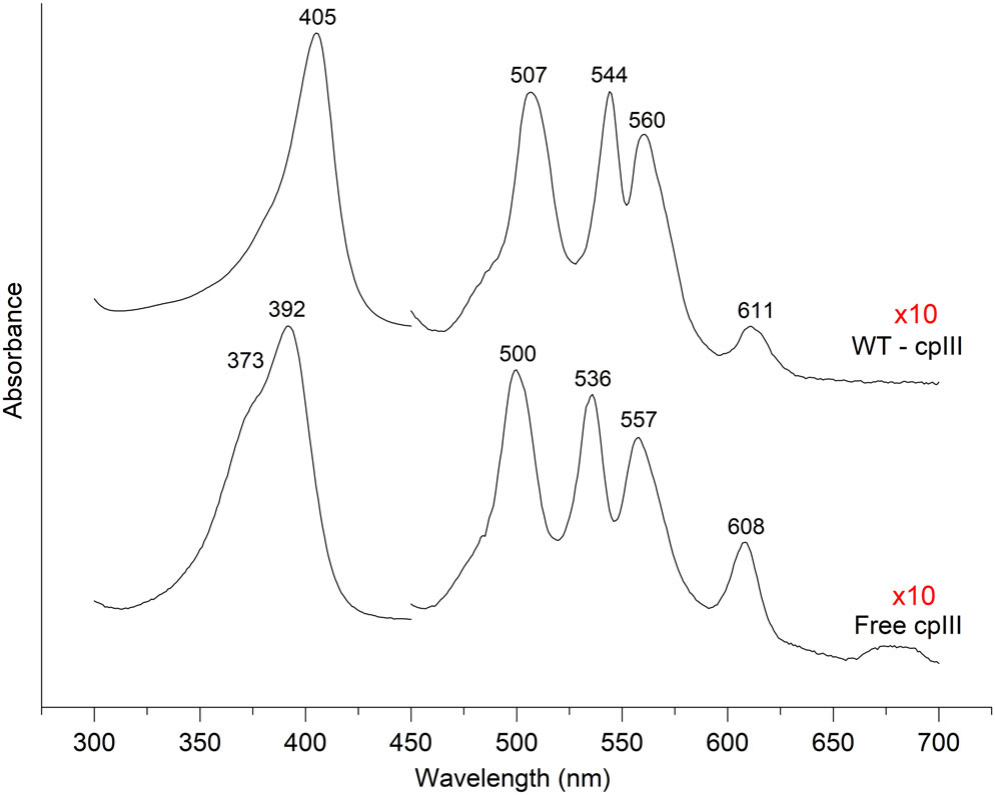
UV–vis electronic absorption spectra of free cpIII (bottom) and cpIII‐*Lm*CpfC WT (top)

The UV–vis and RR spectra of free cpIII and upon binding to *Lm*CpfC are compared in Figure 3. The UV–vis absorption spectral pattern of cpIII, characterized by four Q‐band, are typical of a free‐base porphyrin of D_2h_ symmetry. In detail, the intense Soret band has a maximum at 392 nm with a shoulder at 373 nm and the four weak Q bands in the 500–650 nm region are observed at 500–536 nm (Q_y_), and 557–608 nm (Q_x_).^31^ Insertion of the porphyrin into the apo‐protein (*Lm*CpfC) alters the spectrum, resulting in a sharpening of the Soret band (405 nm) and with Q bands at 507, 544, 560, and 611 nm. Similar changes upon substrate insertion have been also observed in the UV–vis spectra of *Sa*CpfC.^32^ These changes indicate that *Lm*CpfC contributes to the coproporphyrin chemical environment. Furthermore, the UV–vis spectrum confirms that the cpIII is fully bound to the apo‐protein, as there are no bands due to the free cpIII.

**FIGURE 4:**
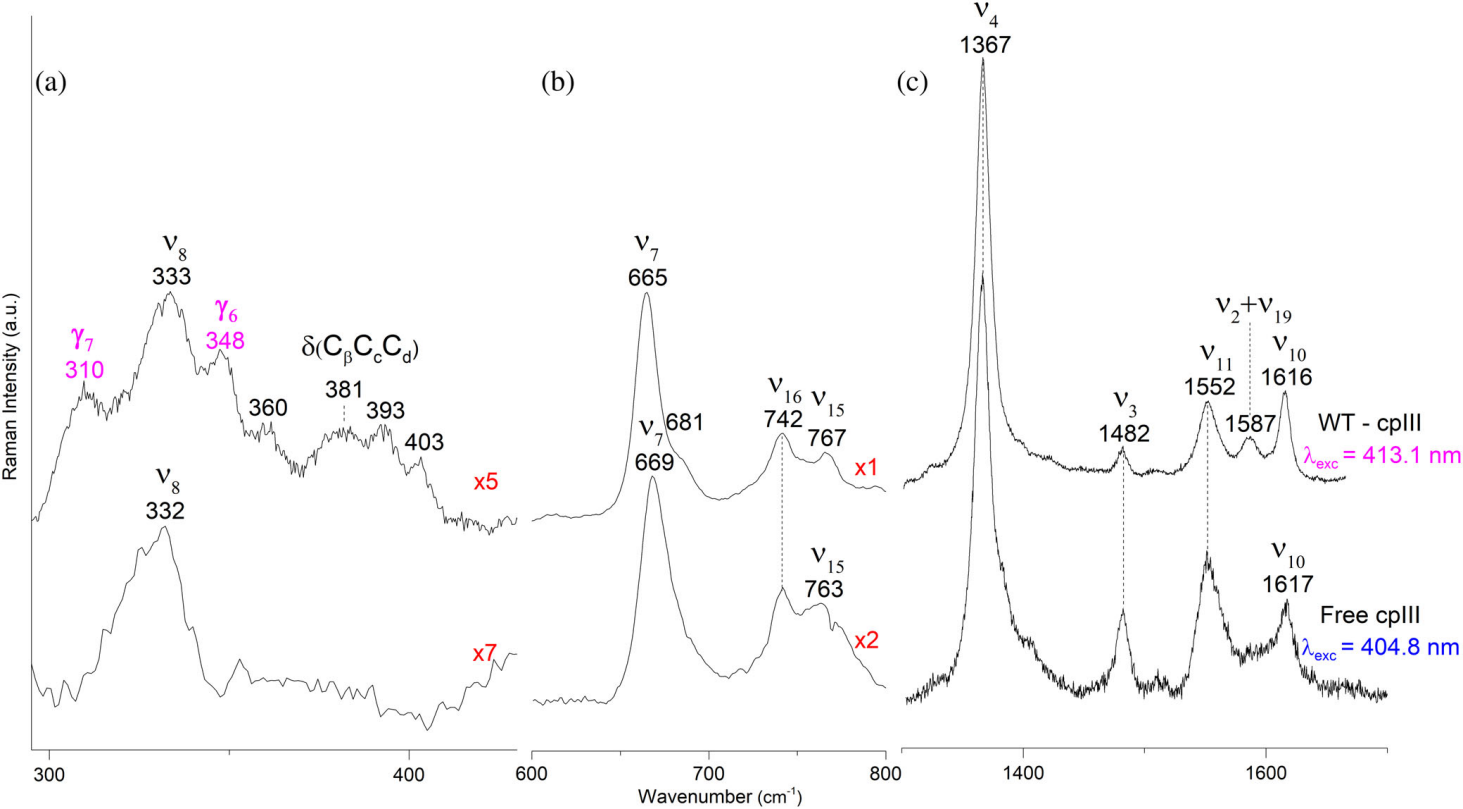
RR spectra in the low (a), middle (b), and high (c) wavenumber regions of free cpIII (bottom) and cpIII‐*Lm*CpfC WT (top). The RR out‐of‐plane band wavenumbers are reported in magenta. RR spectra were measured at low resolution (grating: 1800 grooves mm^−1^) for panel b (lower trace) and panel c, and at high resolution (grating: 3600 grooves mm^−1^) for panel b (upper trace) and panel a. RR, resonance Raman; WT, wild‐type

The Raman spectra in the high wavenumber region obtained in comparable resonance conditions with the Soret band (*λ*_exc_ = 404.8 nm, for the free cpIII, and *λ*_exc_ = 413.1 nm for the bound form) are also very similar (Figure 4c). The core size marker bands are at 1367 (ν_4_), 1482 (ν_3_), 1552 (ν_11_), and 1616–1617 (ν_10_) cm^−1^. In addition, upon binding, the band at 1587 cm^−1^ is intensified. This band is assigned to the overlap of ν_2_ and ν_19_ modes.^33^ These wavenumbers, being very similar to those of the core‐size of a 6cHS metallo‐protein,^28^ are lower than those of the coproheme‐*Lm*CpfC complex, which is a 5cHS species.^17^ In agreement with inverse correlation between the core size marker bands wavenumbers and the size of the core, this suggests that the cpIII‐*Lm*CpfC wild‐type has a larger porphyrin core than the coproheme‐*Lm*CpfC complex.^34^

Instead, in the RR low wavenumber region, insertion of the cpIII into the apo‐protein causes marked changes: in the 300–400 cm^−1^ wavenumber region the out‐of‐plane and propionate bending modes can be identified in the complex (Figure 4a).^24^ The RR spectrum of free cpIII is characterized by a strong band at 332 cm^−1^ (ν_8_), while in the δ(C_β_C_c_C_d_) bending modes of the propionate groups region (360–430 cm^−1^) does not display defined bands. Upon complexation, a band at 333 cm^−1^ (ν_8_) and four propionate δ(C_β_C_c_C_d_) bending modes are clearly observed at 360, 381, 393, and 403 cm^−1^. These data confirm that in solution, as in the crystal phase, the porphyrin is stabilized by the formation of H‐bonds, with different strengths, between polar amino acids and the propionate groups, since the propionate δ(C_β_C_c_C_d_) bending modes wavenumbers are related to the strength and number of hydrogen bonds between the propionate and the amino acids (see below).

Moreover, upon binding of the cpIII into the *Lm*CpfC protein, the activation of the γ_7_ and γ_6_, out‐of‐plane modes (A_2u_ under the D_4h_ symmetry, or B_1u_ under the D_2h_ symmetry) at 310 and 348 cm^−1^, are observed (Figure 4a, top). Since these bands, normally RR‐inactive in a planar system, are activated by the distortion of the tetrapyrrole,^35^ the cpIII binding into the protein results in a doming‐like distortion from planarity of the porphyrin group, as a consequence of the H‐bond interactions of the propionates with the side chains of the pocket. Similar results were obtained for the yeast fC from *Saccharomyces cerevisiae*: the binding of mesoporphyrin free base (MPH_2_) did not affect the enzyme RR spectra, but the ternary complex of MPH_2_ with ferrochelatase and the inhibitor Hg(II) induced a doming distortion of the porphyrin with the activation in the RR spectrum of prominent γ_5_ and γ_6_ out‐of‐plane modes.^36^

### 2.3 Assignment of the propionate bending modes in the RR spectra

**FIGURE 5:**
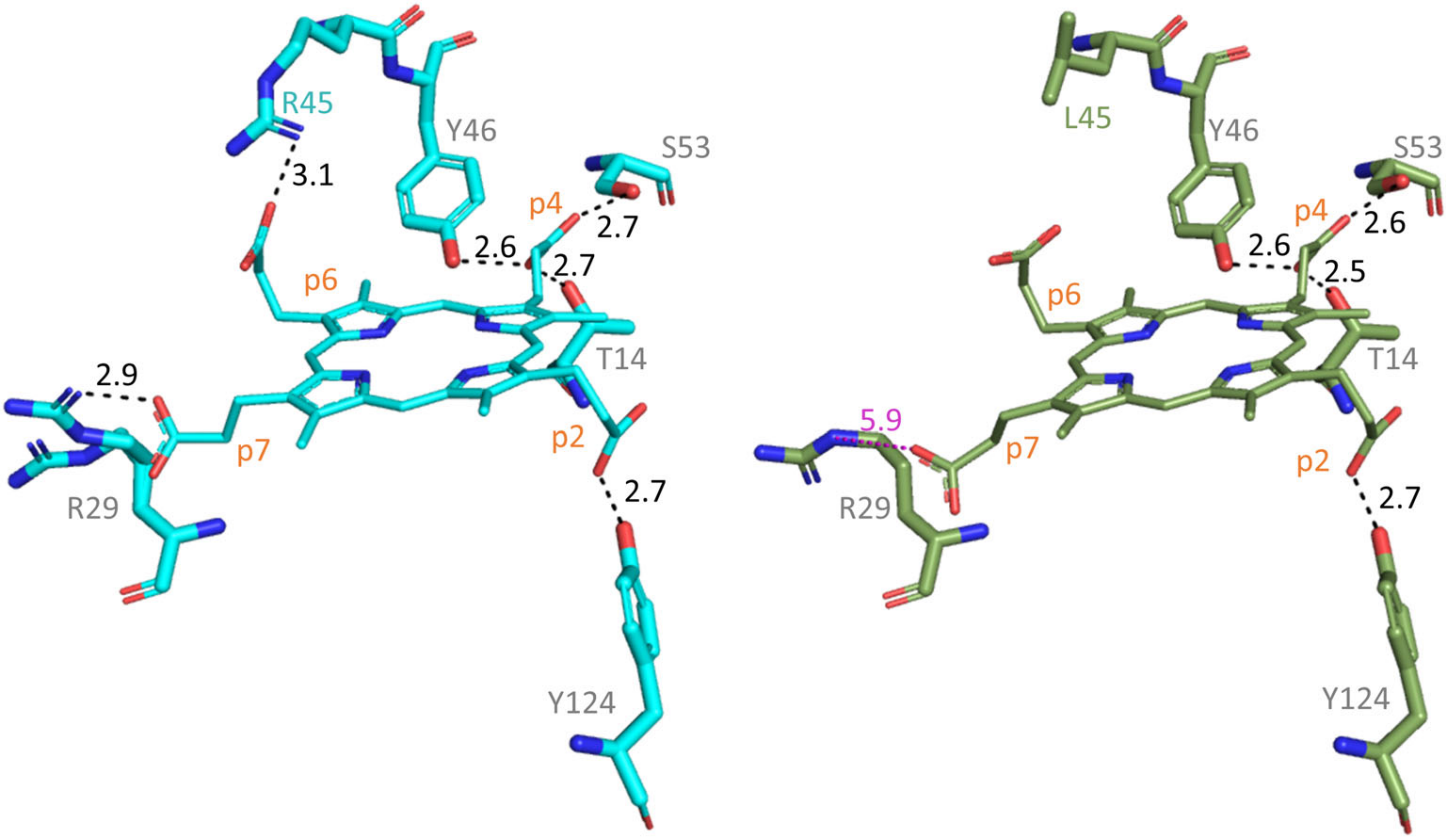
H‐bonding distances between propionates of coproporphyrin III and conserved amino acid residues of *Lm*CpfC wild‐type (cyan sticks) and *Lm*CpfC R45L (olive sticks). Distances (in Å) of potential H‐bonds are shown as black dashed lines and distance too long for H‐bonds as pink dashed line

The results obtained upon insertion of cpIII into the enzyme clearly indicate that the hydrogen‐bonding networks connecting several polar amino acid side chains with the propionate groups are decisive for substrate binding and specificity. Similar to the coproheme‐*Lm*CpfC structure, the cpIII‐*Lm*CpfC structure shows that the propionates p2 and p4, which are missing in protoporphyrin IX, are involved in strong H‐bonding interactions. In particular, p4 interacts with three H‐bonding partners (T14, Y46, S53) and p2 with Y124, while the propionates at positions 6 and 7, also present in protoporphyrin IX, interact with R45 and R29, respectively (Figure 5 and Table 2).

**TABLE 2:** Comparison of the length (in Å) of the H‐bond in the WT and R45L complex with cpIII

	WT	R45L
p7 – R29	2.9	5.9
p6 – R45	3.1	–
p4 – S53	2.7	2.6
p4 – Y46	2.6	2.6
p4 – T14	2.7	2.5
p2 – Y124	2.7	2.7

Abbreviation: WT, wild‐type.

**FIGURE 6:**
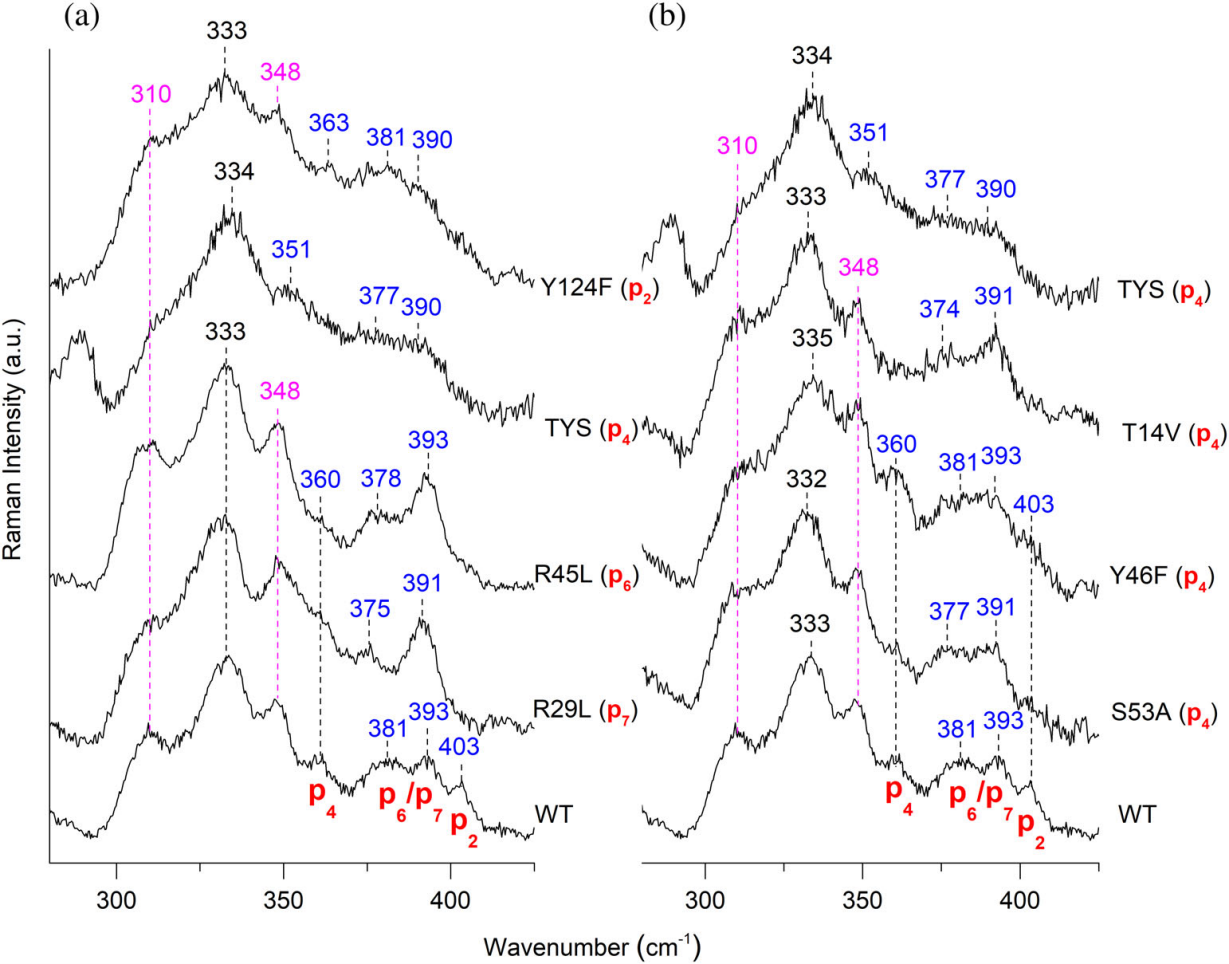
Low wavenumber region RR spectra of cpIII‐*Lm*CpfC variants and WT. RR spectra of the WT and (a) selected variants on p2, p4, p6, and p7, and (b) all the variants on p4 measured at high resolution (grating: 3600 grooves mm^−1^). The propionates, whose H‐bond are removed by mutation together with their assignment are reported in red, in blue their bending modes δ(C_β_C_c_C_d_) wavenumbers, and in magenta the out‐of‐plane band wavenumbers. RR, resonance Raman; WT, wild‐type

In order to evaluate the contribution of the hydrogen bond interactions between the propionates and these residues to the substrate binding, we investigated the cpIII complexes of the WT and several variants by UV–vis and RR spectroscopies to identify the vibrational signatures of the four propionates via their δ(C_β_C_c_C_d_) bending modes. In detail, we have characterized different variants in which H‐bonding interactions with the propionate groups at positions 2, 4, 6, and 7 were selectively eliminated. The investigated variants (Figure S2 and Figure 6) are: the Y124F variant (p2), the T14V, Y46F, S53A single and the T14V/Y46F/S53A (TYS) triple variants (p4), the R45L (p6), and the R29L variants (p7).

The UV–vis and RR (high wavenumber region) spectra of the variants in complex with cpIII hardly show any differences with respect to the WT (Figure S2). On the other hand, as shown in Figure 6, all mutations markedly alter the δ(C_β_C_c_C_d_) bending modes of the propionates, identified in the WT complex by the four well‐defined bands at 360, 381, 393, and 403 cm^−1^. These wavenumbers are related to the strength and number of hydrogen bonds between the propionate and the amino acids. Since stronger hydrogen bonds are correlated to higher wavenumbers,^37^, ^38^, ^39^ by selectively breaking or weakening the H‐bonds by mutation, downshifts of the propionate bending mode wavenumbers are expected, as previously observed.^17^, ^37^

Figure 6a compares the RR spectra of WT and the variants whose H‐bonds with all the propionates have been altered. In the Y124F variant, where the H‐bond involving the p2 is broken, the band at 403 cm^−1^ downshifts giving rise to band at 390 cm^−1^, overlapping with the band at 393 cm^−1^ observed in the WT. Therefore, the band at 403 cm^−1^ is assigned to the bending mode of the p2.

The band at 360 cm^−1^ is assigned to the p4 bending mode, since, being unchanged in the Y124F, R29L, and R45L variants as compared to the WT, is clearly sensitive to the alteration of the H‐bonds involving the p4. The RR spectra of WT together with all the variants in which the H‐bond interactions with p4 were eliminated, are reported in Figure 6b. In the single T14V, and the T14V/Y46F/S53A (TYS) triple variants the 363 cm^−1^ band shifts towards lower wavenumbers, as clearly evidenced in the TYS spectrum by the presence of a weak and broad band at 351 cm^−1^. Similarly to the result obtained for the coproheme complex,^17^ the Y46F variant displays the same propionate bending vibrations as the WT. By contrast, unlike its coproheme complex, also the S53A variant RR spectrum reveals the presence of the 360 cm^−1^ band. Therefore, since this latter residue is H‐bonded to p4(O_2_) (Table 3) the data suggest that the T14–p4(O_1_) interaction primarily determines the strength of the H‐bond network with p4.

**TABLE 3:** Distances (in Å) between the residues and the oxygen atom of the propionates, as determined in the WT cpIII‐*Lm*CpfC (PDB ID: 8AT8) and WT coproheme‐*Lm*CpfC (PDB ID: 6SV3) crystal structures

	WT‐coproporphyrin III					WT‐coproheme				
	p2	p4		p6	p7	p2	p4		p6	p7
	(O_2_)	(O_1_)	(O_2_)	(O_1_)	(O_1_)	(O_2_)	(O_1_)	(O_2_)	(O_1_)	(O_1_)
R29					**2.9**					**2.9**
R45				**3.1**					**2.9**	
S53		4.1	**2.7**				4.0	**2.9**		
Y46		**2.6**	3.6				**2.6**	4.5		
T14		**2.7**	4.5				**2.4**	3.8		
Y124	**2.7**					**3.1**				

*Note*: Values in bold indicate the suggested H‐bonding interactions based on the distances.

The band at 403 cm^−1^, assigned to p2, is also strongly affected by the H‐bond alteration with the p4, since it downshifts in both T14V and TYS variants. This behavior, similar to that previously observed in the p4 variants of the coproheme‐*Lm*CpfC complex, let us to conclude that the H‐bond interactions of p4 and p2 are also coupled in the cpIII‐*Lm*CpfC complex.

Unexpectedly, the RR spectra of the variant involved in the H‐bond with p7 (R29L) and p6 (R45L) are very similar (Figure 6a), both being characterized by three bands, at 360, 375–378, and 391–393 cm^−1^. In both cases the band at 403 cm^−1^ in the WT (p2) downshifts and overlaps with the band at 393 cm^−1^ which, therefore, is markedly intensified as compared to the WT. The similarity between the spectra of R29L and R45L can be rationalized considering the concomitant destabilizing effect on the R29‐p7 interaction by the R45L mutation, where the H‐bond with p6 has been eliminated. The cpIII‐R45L (*Lm*CpfC) crystal structure shows that, in the absence of the R45‐p6 H‐bond, the R29‐p7 H‐bond length changes from 2.9 Å in the WT to 5.9 Å in the R45L mutant (Figure 5, Table 2).

In solution the loss of the p6‐R45 H‐bond causes a dramatic weakening of the whole H‐bond network, since in addition to the rupture of the H‐bond between p7 and R29, it also makes the H‐bond between p2 and Y124 much weaker, as compared to the WT (downshift of the 403 cm^−1^ mode in the WT to 393 cm^−1^ in the mutant). The crystallographic data suggest that p6 and p7 are coupled, and the RR in solution clearly indicate that an overall readjustment of the propionate H‐bonds occurs as well in both R45L and R29L variants.

Therefore, due to a strong coupling between p6 and p7, in the R45L and R29L RR spectra the bands at 375–378 and 391–393 cm^−1^ are ascribed to the p6 and p7 bending modes, although a conclusive selective assignment is not possible in this case. In fact, the 6 cm^−1^ downshift of the band observed at 381 cm^−1^ in the R29L RR spectrum might suggest that this band corresponds to the p7 bending mode. However, the hydrogen bond network between the protein and the substrate or the product do not differ largely (Table 3), and the same assignment of the peripheral substituents bending modes between cpIII and coproheme cannot be ruled out. For the reader's convenience, we report that in the WT coproheme‐*Lm*CpfC the bands at 363, 374, 385, and 402 cm^−1^ were assigned to the bending modes of p4, p6, p7, and p2, respectively.^17^

The presence of all the four‐propionate side‐chains seems to be essential for the interaction with the cpIII substrate, whereas in the coproheme‐*Lm*CpfC complex p2 and p4 were dominant. However, the RR data highlight that the strong H‐bonds between the residues and p4 are responsible for the distortion of the porphyrin ring, since the out‐of‐plane modes observed at 310 and 348 cm^−1^ almost disappeared in the triple mutant. Therefore, the H‐bonds involving p4 are fundamental for binding, stability, and correct orientation of the substrate in the active site pocket, in agreement with previous observations on thermostability, binding kinetics, and iron insertion ability of the *Lm*CpfC native protein and variants.^17^

## 3 DISCUSSION

The present results highlight that all four propionate side‐chains are essential requirements for the correct orientation and stabilization of the cpIII inside the active site, confirming the substrate specificity of *Lm*CpfC towards the four propionates.^17^ Unlike in the coproheme‐*Lm*CpfC complex, not only the p2 and p4 H‐bonds are coupled, but also the alterations of the H‐bond interactions of the p6 and p7 are reciprocally influenced. Moreover, multiple strong H‐bonds established between the polar residues and the p4 are responsible for the distortion of the porphyrin ring, since the γ_6_ and γ_7_ out‐of‐plane modes markedly lose intensity in the triple TYS variant. In the coproheme‐*Lm*CpfC there is of course a strong additional stabilizing contribution from the central ferric iron to the proximal Y12 residue, which is naturally lacking in the cpIII‐*Lm*CpfC complex.^17^

The use of the physiological substrate (with four propionates) for a monoderm bacterial fC clearly indicates that all studies on *Bacillus subtilis* CpfC (*Bs*CpfC), using as substrate either PPIX or the two‐propionate model substrates, N‐MeMP and dSDP, gave rise to misleading data. These porphyrins do not enter the cleft as deeply as coproheme or cpIII in *Lm*CpfC,^10^, ^11^, ^16^ and, therefore, N‐MeMP ‐bound *Bs*CpfC, cannot be taken as a reference model for bacterial ferrochelatases. In particular, unlike in the cpIII‐*Lm*CpfC complex, the structure of N‐MeMP ‐bound *Bs*CpfC complex shows that the pyrrole rings C and D and their propionic side‐chains (p6 and p7) are extended outwards, while only rings A and B (with the vinyl groups) are positioned in the interior of the protein.^4^, ^10^, ^40^ In addition, the structures with and without *N*‐MeMP do not show any difference in the orientation of the active site amino acids or in the overall active site pocket shape.^10^ A high degree of selectivity for a particular porphyrin has also been observed in the membrane‐associated human and murine ferrochelatases. In both enzymes the two porphyrin propionates, p6 and p7, interact with active site residues via hydrogen and ionic bonds to ensure a highly specific spatial orientation of the porphyrin in the active site.^14^, ^41^ Moreover, in the murine PpfC, the interaction between the vinyl groups (v2 and v4) and the active site loop, most likely plays a determinant role in the porphyrin substrate specificity.^18^, ^41^ In fact, alterations in the vibrational modes of the vinyl and propionate groups were observed in some variants, suggesting that the reorientation and relocation of the macrocycle can occur in proteins with mutated active site conserved residues.^41^ Furthermore, in the RR spectra of the murine PpfC a sharpening of the vinyl stretching mode ν(C=C) was taken as an indication of a possible movement and orientation restriction of the vinyl groups within the protein environment upon porphyrin binding.^41^, ^42^, ^43^

Distortion of the macrocycle is one of the factors that may control the chemical properties of porphyrins in living organisms^22^, ^44^ and was found of particular relevance for ferrochelatases.^45^

As mentioned above, in solution the binding of the cpIII ring into the active site of *Lm*CpfC and the formation of the H‐bond interactions between the propionates and the side chains cause a doming‐like deformation of the porphyrin ring, consistent with the enhancement of the γ_6_ and γ_7_, out‐of‐plane modes. Similarly, a porphyrin distortion was observed in the RR spectrum of the free‐base protoporphyrin IX bound to wild‐type murine ferrochelatase (PpfC). In that case, however, the observation of the γ_15_ out‐of‐plane mode (B_2u_ symmetry under D_4h_ group or B_1u_ symmetry under D_2h_ group), around 700 cm^−1^ (not observed in the present work, see Figure 4b), led to the conclusion that a saddling‐like out‐of‐plane deformation took place.^46^

Up to now, many RR studies have studied the conformational changes that occur to the substrate on binding to fC and metalation mechanism in murine fCs, *Saccharomyces cerevisiae* fCs, catalytic antibodies, and *Bs*CpfC.^36^, ^42^, ^46^, ^47^, ^48^, ^49^ The results, all show that saddling is the predominant porphyrin out‐of‐plane deformation induced upon binding to ferrochelatase. Based on the structure of the *N*‐MeMP transition state analog bound to *Bs*CpfC taken as reference model, the preponderance of saddling distortion observed upon binding of the free substrate into the protein, has been considered particularly relevant to initiate the fC mechanism. This out‐of‐plane deformation exposes both the protons and the lone electron pairs of the nitrogen atoms of the porphyrin core, facilitating the metal insertion.^50^, ^51^, ^52^ This porphyrin deformation was further investigated by site‐directed mutagenesis in murine ferrochelatase. In fact, the variants with a low catalytic efficiency also exhibited a decrease in the intensity of RR out‐of‐plane vibrational mode γ_15_ mode, suggesting that the initial PPIX saddling deformation could be directly correlated with catalytic affinity and in particular with metal selectivity.^43^, ^45^ However, since the X‐ray structure of human‐PpfC clearly showed that the bound porphyrin macrocycle was only modestly distorted, this previous conclusion might need to be reevaluated.^14^

In this work it is evident that the porphyrin macrocycle of the cpIII‐*Lm*CpfC complex also exhibits an out‐of‐plane distortion, in contrast to the planar product‐bound (coproheme‐) *Lm*CpfC ring.^16^ However, the type of distortion is different when considering the crystal or the solution data. In the crystal, a saddling‐like distortion is evidenced when comparing the porphyrins from cpIII‐*Lm*CpfC and coproheme‐*Lm*CpfC wild‐type complexes (Figure 2), since the tetrapyrrole ring of cpIII adopts a saddling distortion, as described above. On the other hand, in the RR solution spectra the γ_15_ saddling mode is absent (Figure 4b) and only the out‐of‐plane modes characteristic for a doming‐like distortion are activated (Figure 4a). A difference between solution and crystal is not so surprising. It is well known that both crystallization and X‐ray acquisition may alter or damage a protein structure or influence the state of a co‐factor.^53^, ^54^, ^55^, ^56^ In many cases, the comparison of crystal and in solution heme protein spectra using micro‐RR technique has evidenced differences in the spin and coordination state of the heme iron.^57^, ^58^, ^59^, ^60^, ^61^, ^62^ In addition, crystallization may stabilize the lowest soluble protein conformation, which is usually not greatly populated in solution. This has been observed for protein structures with a very flexible architecture in the proximity of the heme cavity, such as truncated hemoglobin from the Antarctic marine bacterium *Pseudoalteromonas haloplanktis* TAC125.^63^ We cannot, therefore, exclude that the apparent inconsistency on the type of distortion, as highlighted by RR in solution and in the crystal by the X‐ray structure, derives from the different packing forces acting in the two phases. As an example, the heme containing peroxidases belonging to the plant peroxidase superfamily gave rise to similar results. The X‐ray structures of cytochrome *c* peroxidase and *Coprinus cinereus Arthomices ramosus* peroxidases^64^, ^65^, ^66^ show a highly distorted heme group with a strong saddling deformation, induced by the van der Waals contacts, the hydrogen bonding to the propionates, and the interactions with the axial ligand.^67^ However, their RR solution spectra do not show a γ_15_ mode, but only the γ_7_ and γ_6_ out‐of‐plane modes.^68^, ^69^

Our data, together with those obtained for the human fC, suggest the extent of substrate distortion prior to the metal insertion may not influence the catalytic efficiency of fC towards the porphyrin substrate. Thus, we propose that the porphyrin deformation observed upon binding to the apo‐protein is the outcome of the characteristic interactions between the involved substrate and protein moiety and that the proposed further saddling distortion predicted to occur upon metalation is a successive, but not necessarily correlated, step. This conclusion agrees with our previous findings that all the investigated *Lm*CpfC variants are able to insert ferrous iron into cpIII, including the triple variant (TYS), which clearly shows a marked loss of ring deformation in solution, but reaches coproheme saturation, even if at higher ferrous iron concentrations than the other samples.^17^ In agreement with our conclusion is also the RR study of a porphyrin interacting both with an antibody, that catalyzes metal insertion, and the yeast fC enzyme. The results showed that the enzyme‐induced distortion was different from that caused by the antibody. In fact, the antibody bound to the mesoporphyrin free base (MPH_2_) produced the saddling distortion with the activation of the γ_15_ out‐of‐plane mode, similar to the bound N‐MeMP porphyrin ring. Insertion of MPH_2_ into yeast fC, however, caused minor spectral changes indicating a little influence from the protein. On the contrary, binding of the inhibitor Hg(II) to MPH_2_‐fC complex induced a doming distortion. Based on this observation the authors suggested an allosteric mechanism, in which a conformational change, which distorts the porphyrin towards the transition state geometry, is induced by metal binding at an adjacent site.^36^

## 4 CONCLUSIONS

In summary, we present for the first time comprehensive and in‐depth spectroscopic and crystallographic studies on monoderm bacterial CpfCs in complex with its physiological porphyrin substrate (cpIII). We show that binding and substrate specificity is directly related to the hydrogen bond interactions between the four propionate groups and the protein matrix. Their RR bending modes assigned with the help of specific site‐directed variants, confirm the presence of hydrogen bonds with different strengths in solution, which agrees with the crystal data. The metal free tetrapyrrole is clearly distorted when bound to the wild‐type protein, prior to insertion of ferrous iron, as a consequence of the interactions of the protein moiety with all four propionate groups. Therefore, the environment of the active site controls, to a great extent, the orientation and deformation of the porphyrin, and the distortion is not necessarily a mandatory pre‐condition for iron insertion. Future studies will target the relevance and prevalence of porphyrin distortion during the actual iron incorporation process.

## 5 MATERIALS AND METHODS

### 5.1 Expression and purification of *Lm*CpfC wild‐type and variants

*Lm*CpfC wild‐type and variants were generated, expressed, and purified as reported previously.^17^ Prior to measurements or drop setting for crystallization the reconstituted cpIII‐*Lm*CpfC complex was purified to monodispersity using a size exclusion chromatography column (Superdex 200 16/600, Cytiva). All samples were additionally checked for purity and monomeric state using an HPLC (Shimadzu prominence LC20)‐SEC (Superdex 200 10/300, Cytiva)‐MALS (Wyatt, Heleos Dawn8+) system.

### 5.2 Sample preparation for UV–vis and RR experiments

Coproporphyrin III was purchased from Frontiers Scientific (product number: C654‐3) as lyophilized powder and then dissolved in 0.5 M NaOH. The free cpIII sample was prepared by diluting the cpIII solution in 50 mM HEPES buffer pH 7.4 to obtain a concentration in the range of 5–10 μM. The sample concentration was determined using an extinction coefficient for free cpIII of 150,736 M^−1^ cm^−1^ at 393 nm.^17^ The protein samples were prepared by adding the cpIII solution to a solution of the apoprotein–*Lm*CpfC dissolved in 50 mM HEPES buffer pH 7.4. The cpIII:apoprotein ratios (Table S1) were determined for the wild‐type protein and its variants by UV–vis spectroscopy titration to ensure the complete binding of the substrate, and the absence of an excess of free porphyrin. The sample concentration, in the range of 18–40 μM, were determined using an extinction coefficient of 93,594 M^−1^ cm^−1^ at 393 nm of bound cpIII to the wild‐type protein. This extinction coefficient is deduced from the ratio of the absorption values of the Soret maxima from free cpIII and the fully cpIII‐bound *Lm*CpfC wild‐type solutions. Experimentally we started with a known concentration of free cpIII in the cuvette and titrated small volumes (max. 5 μL in 1000 μL) of highly concentrated *Lm*CpfC to the solution until the spectrum did not change anymore and cpIII complete binding to *Lm*CpfC was guaranteed.

### 5.3 UV–vis electronic absorption spectroscopy

UV–vis electronic absorption spectra were recorded using a 1 mm cuvette or a 5 mm NMR tube at 25°C by means of a Cary 60 spectrophotometer (Agilent Technologies, Santa Clara, CA) with a scan rate of 600 nm min^−1^ and a resolution of 1.5 nm. All the spectra were normalized to the intensity of the Soret band and the 450–700 nm region has been further magnified by a factor indicated in the figures, depending on the considered sample.

### 5.4 Resonance Raman spectroscopy

RR spectra of samples placed in a slowly rotating 5 mm NMR tube were obtained at room temperature by excitation with the 404.8 nm lines of a MatchBox Series diode laser (Integrated Optics, Vilnius, Lithuania) and the 413.1 nm lines of an Innova300C Kr^+^ laser (Coherent, Santa Clara, CA). The choice of the excitation wavelength of 413.1 nm for the WT‐cpIII complex depends on the low stability of the sample upon irradiation in complete resonance with the Soret band (i.e., at 404.8 nm). The laser power at the sample was 2.0 mW for the free cpIII and 1.0 mW for the protein samples. Back‐scattered light was collected and focused into a triple spectrometer (Acton Research, Acton, MA), consisting of two SpectraPro 2300i instruments working in the subtractive mode and, in the final stage, a SpectraPro 2500i instrument equipped with a grating of 3600 or 1800 grooves mm^−1^ and a liquid nitrogen‐cooled charge‐coupled device (CCD) detector (Roper Scientific Princeton Instruments). Based on the optical properties of the spectrometer a spectral resolution of 1.2 cm^−1^ for the 3600 grooves mm^−1^, and 4 cm^−1^ for the 1800 grooves mm^−1^ was calculated. The RR spectra were calibrated using indene and carbon tetrachloride as standards to an accuracy of 1 cm^−1^ for intense isolated bands.

To ensure reproducibility all the RR measurements were repeated several times under the same conditions. To guarantee that no degradation of the sample occurred under the experimental conditions the UV–vis electronic absorption spectra were recorded both before and after RR measurements. To improve the signal‐to‐noise ratio the RR spectra were summed, if no spectral differences were noted. Table S2 summarizes the integration time and the number of averaged spectra reported in the figures. All the spectra were baseline‐corrected, normalized to the intensity of the ν_4_ band (1367 cm^−1^) and to the ν_8_ band (332–335 cm^−1^) for the high and low wavenumber region, respectively, and shifted along the ordinate axis, in the figures, for a better visualization.

### 5.5 Crystallization

*Lm*CpfC wild‐type in complex with cpIII crystallized in 17.455% (w/v) PEG MME 2K, 0.1 M BIS‐TRIS pH 6.3, and 0.2 M calcium acetate. The condition was found by screening for optimized conditions, by varying the concentrations of all components in a systematic manner (PEG MME 2K: 15%–25%, BIS‐TRIS: 0.1 M pH 5.8–6.3, calcium acetate: 0.1–0.4 M). Optimization screens were prepared using the liquid handling robot FORMULATOR 10 (Formulatrix). The drops where set using MOSQUITO LCP (ttplabtech) in MRC three‐well plates (SWISSCI). One drop contained 150 nL cpIII‐*Lm*CpfC wild‐type (250 μM) and 150 nL of the mother liquor. Crystals (plate shaped) with the dimensions of 50 × 20 × 5 μm formed within 4 days. Crystals were flash‐vitrified in liquid nitrogen using the mother liquor containing 20% glycerol as cryo‐protectant.

*Lm*CpfC R45L in complex with cpIII crystallized in 18% (w/v) PEG 8K and 20% glycerol with a 1:1000 dilution of apo wild‐type germination solution containing crushed apo wild‐type crystals and 5 mM phosphate buffer. The equipment used was the same as for wild‐type. One drop contained 150 nL of cpIII‐R45L variant (350 μM), 200 nL of mother liquor, and 30 nL of 1:1000 seed solution. Crystals (small cubic shapes) with dimensions 20 × 20 × 20 μm formed within 5 days. The crystals were flash‐vitrified in liquid nitrogen directly from the mother liquor, as it already contained 20% glycerol.

### 5.6 Data collection and refinement

Data for both data sets were collected at the European Synchrotron Radiation Facility (Grenoble, France). Data from the *Lm*CpfC wild‐type in complex with cpIII were collected at the Massif‐3 beamline at 100 K using a DECTRIS EIGER X 4M detector. The autoproc pipeline was used to process the data set. Data from the R45L variant in complex with cpIII were acquired at beamline ID23‐1 at 100 K using a DECTRIS PILATUS 6M F detector. The XIA2‐dials pipeline was used to process the data set.

Data quality assessment for both datasets was performed using Xtriage. For both, the phase problem was solved by molecular replacement with Phaser‐MR^70^ using the pdb structure 6RWV of apo‐*Lm*CpfC. The initial models were generated with AUTOBUILD^71^ and further improved by iterative cycles of manual model generation with COOT^72^ and maximum likelihood refinement with PHENIX‐refine.^73^ The algorithm of French and Wilson^74^ was used by PHENIX‐refine to convert intensities to amplitudes. Restraints for cpIII (ligand ID now HT9) were obtained by downloading the Mol file for cpIII from the KEGG compound database and generating the restraint file for refinement in CCP4.^75^ Finalization of the refinements included translation release screw (TLS) parameters, automatic addition of hydrogen and water molecules, optimization of the isotropic B‐factor model for X‐ray/stereochemical weight, and optimization of X‐ray/ADP weight. The model was validated using MolProbity.^76^ Images were generated using PyMOL (http://www.pymol.org).

## STRUCTURE COORDINATES

Coordinates for the cpIII‐*Lm*CpfC wild‐type and cpIII‐*Lm*CpfC R45L are deposited in the Protein Data Bank (www.pdb.org) and can be found with the accession PDB ID: 8AT8 and 8AW7, respectively.

## AUTHOR CONTRIBUTIONS

**Andrea Dali:** Data curation (equal); formal analysis (equal); investigation (equal); methodology (equal); visualization (equal); writing – original draft (equal); writing – review and editing (equal). **Thomas Gabler:** Data curation (equal); formal analysis (equal); investigation (equal); methodology (equal); visualization (equal); writing – original draft (equal); writing – review and editing (equal). **Federico Sebastiani:** Data curation (equal); formal analysis (equal); investigation (equal); methodology (equal); visualization (equal); writing – original draft (equal); writing – review and editing (equal). **Alina Destinger:** Data curation (equal); formal analysis (supporting). **Paul Furtmüller:** Project administration (supporting); resources (equal); supervision (supporting); writing – review and editing (equal). **Vera Pfanzagl:** Data curation (equal); formal analysis (equal); writing – review and editing (equal). **Maurizio Becucci:** Conceptualization (supporting); formal analysis (equal); investigation (equal); project administration (supporting); supervision (equal); writing – original draft (equal); writing – review and editing (equal). **Giulietta Smulevich:** Conceptualization (equal); formal analysis (equal); funding acquisition (supporting); investigation (equal); methodology (equal); project administration (equal); resources (equal); supervision (equal); writing – original draft (equal); writing – review and editing (equal). **Stefan Hofbauer:** Conceptualization (equal); formal analysis (equal); funding acquisition (lead); investigation (equal); project administration (equal); resources (equal); supervision (equal); visualization (equal); writing – original draft (equal); writing – review and editing (equal).

## Supporting information


**Figure S1.** Overview of relevant porphyrin substrates and products. Pyrrole rings are labeled in blue and porphyrin substituent positions in orange
**Figure S2.** UV–vis electronic absorption spectra (A) and high wavenumber region RR spectra (B) of cpIII‐*Lm*CpfC WT and variants. In red propionates, whose H‐bond are broken by mutation, are reported. The 450–700 nm region of the UV–vis spectra has been magnified by a factor from 15‐ to 40‐fold, depending on the considered sample
**Table S1.** Coproporphyrin III: apoprotein ratio utilized for all the variants and the WT
**Table S2.** Integration time and number of averaged RR spectra (average/integration time) for the free cpIII and cpIII‐*Lm*CpfC complexes of WT and variants, obtained with low (grating: 1800 grooves mm^−1^) and high resolution (grating: 3600 grooves mm^−1^) with different excitation wavelengths
