# Supplementary material for: Active site architecture of coproporphyrin ferrochelatase with its physiological substrate coproporphyrin III: Propionate interactions and porphyrin core deformation
Source: Protein Sci. 2023 Jan 1;32(1):e4534. doi: 10.1002/pro.4534 (PMC9794026; doi:10.1002/pro.4534)
Supplement: Supplementary file 1 — Figure S1. Overview of relevant porphyrin substrates and products. Pyrrole rings are labeled in blue and porphyrin substituent positions in orange Figure S2. UV–vis electronic absorption spectra (A) and high wavenumber region RR spectra (B) of cpIII‐LmCpfC WT and variants. In red propionates, whose H‐bond are broken by mutation, are reported. The 450–700 nm region of the UV–vis spectra has been magnified by a factor from 15‐ to 40‐fold, depending on the considered sample Table S1. Coproporphyrin III: apoprotein ratio utilized for all the variants and the WT Table S2. Integration time and number of averaged RR spectra (average/integration time) for the free cpIII and cpIII‐LmCpfC complexes of WT and variants, obtained with low (grating: 1800 grooves mm−1) and high resolution (grating: 3600 grooves mm−1) with different excitation wavelengths [file PRO-32-e4534-s001.docx]

**Supporting Information**

**Active site architecture of coproporphyrin ferrochelatase with its physiological substrate coproporphyrin III: propionate interaction and porphyrin distortion.**

Andrea Dali^$1^, Thomas Gabler^$2^, Federico Sebastiani^$1^, Alina Destinger^2^, Paul Georg Furtmüller^2^, Vera Pfanzagl^2^, Maurizio Becucci^1^*, Giulietta Smulevich^1,3^*, and Stefan Hofbauer^2^*

^1^Dipartimento di Chimica “Ugo Schiff”- DICUS, Università di Firenze, Via della Lastruccia 3-13, I-50019 Sesto Fiorentino (FI), Italy

^2^University of Natural Resources and Life Sciences, Vienna, Department of Chemistry, Institute of Biochemistry, Muthgasse 18, A-1190 Vienna, Austria

^3^INSTM Research Unit of Firenze, Sesto Fiorentino (Fi), Italy

^$^ These authors contributed equally

Corresponding Authors

*e-mail: maurizio.becucci@unifi.it, phone: +39-055-4573089

*e-mail: giulietta.smulevich@unifi.it, phone: +39-055-4573083

*e-mail: stefan.hofbauer@boku.ac.at, phone: +43-1-47654-77258

**S1 Supporting Figures**

**S2 Supporting Tables**

**S1 Supporting Figures**

**
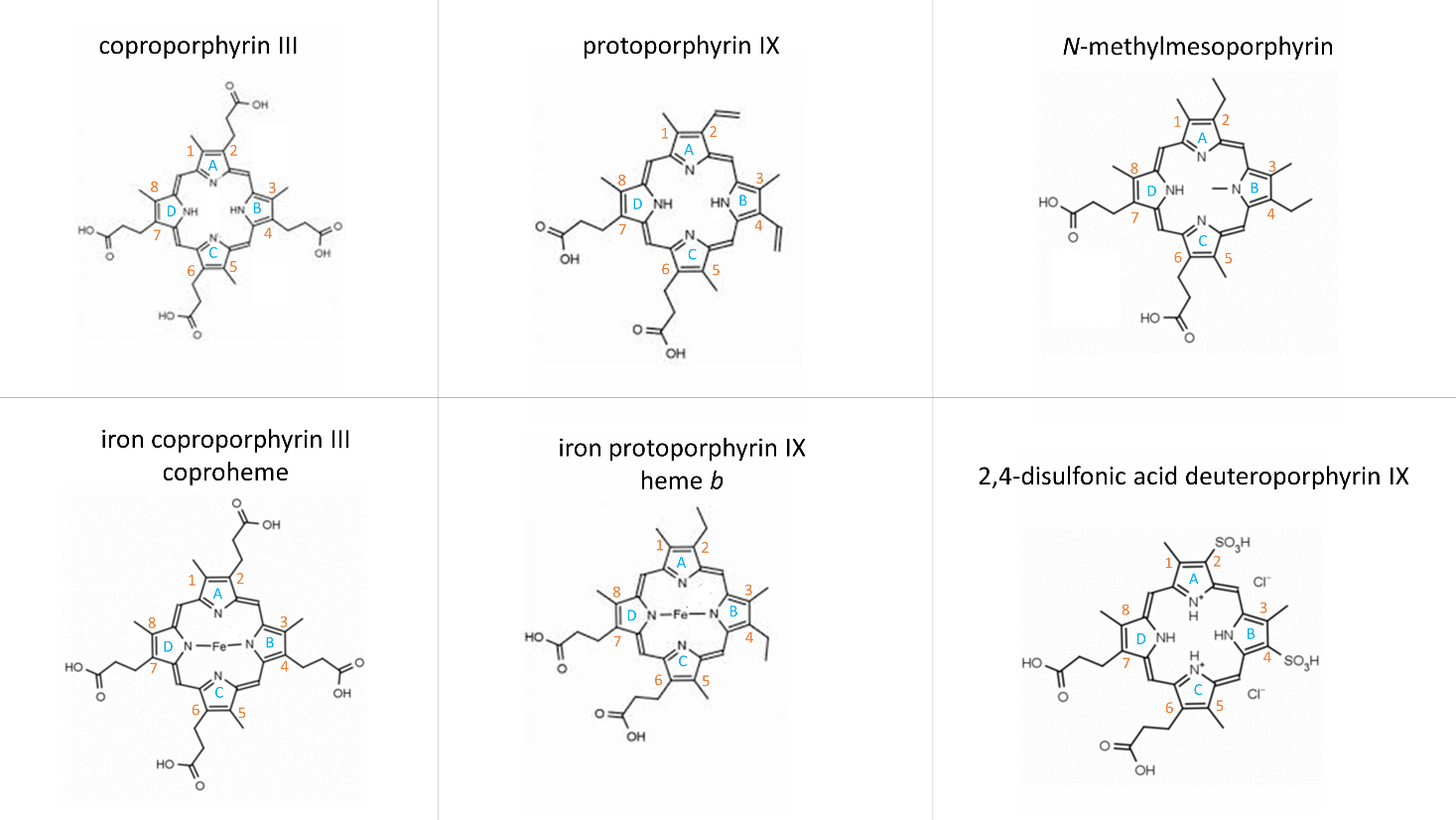
**

**Figure S1.** Overview of relevant porphyrin substrates and products. Pyrrole rings are labeled in blue and porphyrin substituent positions in orange.


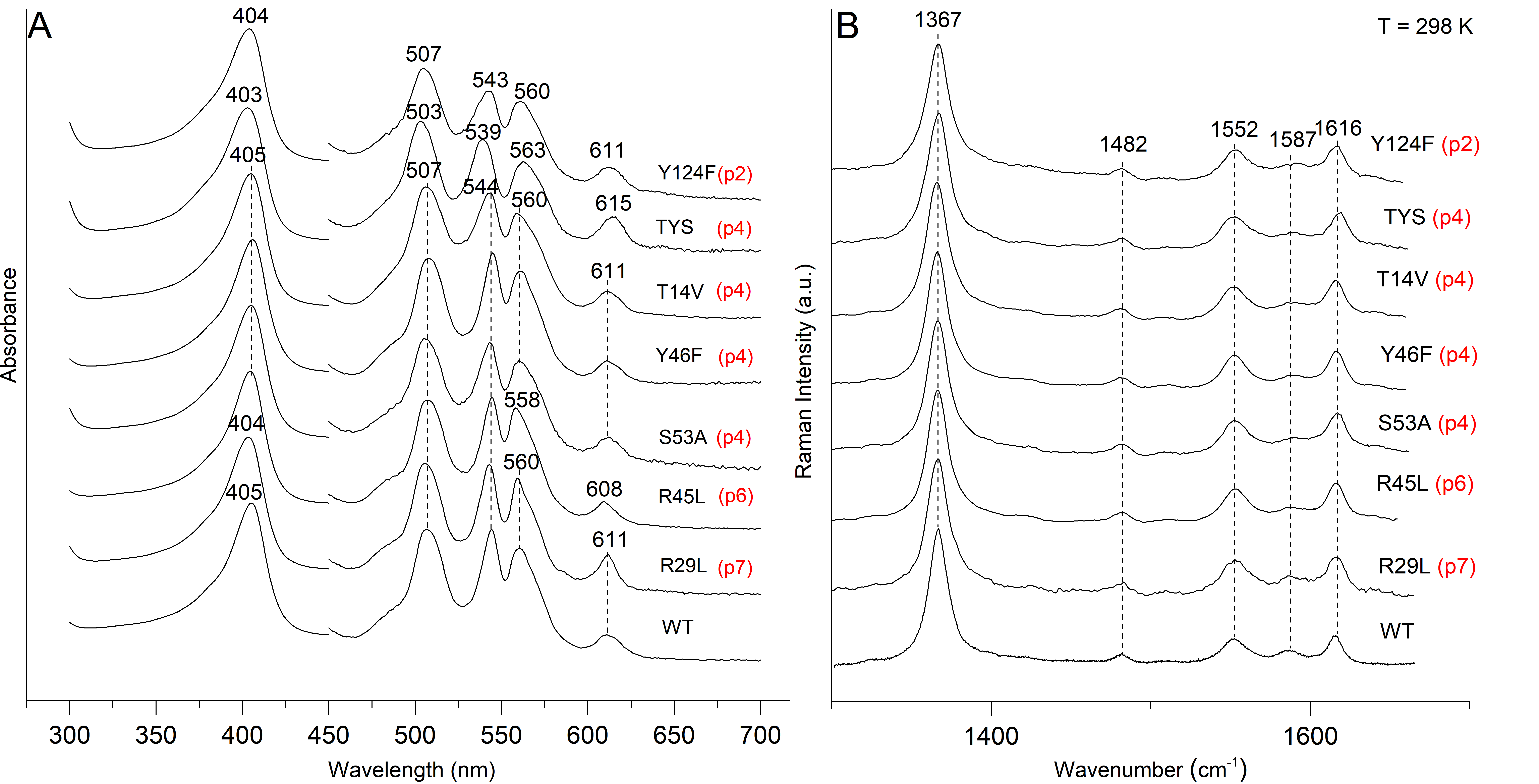


**Figure S2.** UV-vis electronic absorption spectra (A) and high wavenumber region RR spectra (B) of cpIII – *Lm*CpfC WT and variants. In red propionates, whose H-bond are broken by mutation, are reported. The 450-700 nm region of the UV-vis spectra has been magnified by a factor from 15- to 20-fold, depending on the considered sample.

**S2 Supporting Tables**

**Table S1**. Coproporphyrin III : apoprotein ratio utilized for all the variants and the WT.

| Sample | Coproporphyrin III : apo-protein ratio |
| --- | --- |
| WT | 1 : 1.3 |
| p7 – R29L | 1 : 3 |
| p6 – R45L | 1 : 1.7 |
| p4 – S53A | 1 : 3 |
| p4 – Y46F | 1 : 1.2 |
| p4 – T14V | 1 : 1.7 |
| p4 - TYS | 1 : 4 |
| p2 – Y124F | 1 : 4 |

**Table S2**. Integration time and number of averaged RR spectra (average/integration time) for the free cpIII and WT and variants cpIII – *Lm*CpfC complexes, obtained with low (grating: 1800 grooves/mm) and high resolution (grating: 3600 grooves/mm) with different excitation wavelengths.

|  | | Low resolution | High resolution | |
| --- | --- | --- | --- | --- |
| Sample | Excitation wavelenght |  | Low frequency region | High frequency region |
| Free cpIII | 404.8 nm | 64 spectra / 320 min | - | 2 spectra / 10 min |
| CpIII – WT | 413.1 nm | - | 50 spectra / 250 min | 16 spectra / 80 min |
| CpIII – R29L | 413.1 nm | 10 spectra / 10 min | 32 spectra / 160 min | - |
| CpIII – R45L | 413.1 nm | 35 spectra / 35 min | 56 spectra / 280 min | - |
| CpIII – S53A | 413.1 nm | 8 spectra / 40 min | 38 spectra / 190 min | - |
| CpIII – Y46F | 413.1 nm | 35 spectra / 35 min | 50 spectra / 250 min | - |
| CpIII – T14V | 413.1 nm | 40 spectra / 40 min | 32 spectra / 160 min | - |
| CpIII – TYS | 413.1 nm | 35 spectra / 60 min | 74 spectra / 370 min | - |
| CpIII – Y124F | 413.1 nm | 35 spectra / 60 min | 52 spectra / 260 min | - |
